# Role of the Endocannabinoid System in the Adipose Tissue with Focus on Energy Metabolism

**DOI:** 10.3390/cells10061279

**Published:** 2021-05-21

**Authors:** Volatiana Rakotoarivelo, Jyoti Sihag, Nicolas Flamand

**Affiliations:** 1 Centre de recherche de l’Institut universitaire de cardiologie et de pneumologie de Québec, Département de médecine, Faculté de médecine, Université Laval, Québec City, QC G1V 4G5, Canada; Volatiana.Rakotoarivelo@criucpq.ulaval.ca (V.R.); jyoti.sihag@criucpq.ulaval.ca (J.S.); 2 Canada Excellence Research Chair on the Microbiome-Endocannabinoidome Axis in Metabolic Health (CERC-MEND), Université Laval, Québec City, QC G1V 4G5, Canada; 3 Faculty of Agriculture and Food Sciences, Université Laval, Québec City, QC G1V 0A6, Canada; 4 Department of Foods and Nutrition, Chaudhary Charan Singh Haryana Agricultural University, Hisar 125004, India

**Keywords:** endocannabinoid, white adipose tissue, brown adipose tissue, N-arachidonoyl-ethanolamine (AEA), 2-arachidonoyl-glycerol (2-AG), energy metabolism

## Abstract

The endocannabinoid system is involved in a wide range of processes including the control of energy acquisition and expenditure. Endocannabinoids and their receptors are present in the central nervous system but also in peripheral tissues, notably the adipose tissues. The endocannabinoid system interacts with two main hormones regulating appetite, namely leptin and ghrelin. The inhibitory effect of the cannabinoid receptor 1 (CB_1_) antagonist rimonabant on fat mass suggested that the endocannabinoid system can also have a peripheral action in addition to its effect on appetite reduction. Thus, several investigations have focused on the peripheral role of the endocannabinoid system in the regulation of metabolism. The white adipose tissue stores energy as triglycerides while the brown adipose tissue helps to dissipate energy as heat. The endocannabinoid system regulates several functions of the adipose tissues to favor energy accumulation. In this review we will describe the presence of the endocannabinoid system in the adipose tissue. We will survey the role of the endocannabinoid system in the regulation of white and brown adipose tissue metabolism and how the eCB system participates in obesity and metabolic diseases.

## 1. Introduction

The endocannabinoid (eCB) system comprises the cannabinoid receptors CB_1_ and CB_2_, their lipid based ligands, *N*-arachidonoyl-ethanolamine (AEA) and 2-arachidonoyl-glycerol (2-AG), as well as the enzymes implicated in their metabolism [1]. The eCB system plays a critical role in energy balance, notably at the level of fat accumulation and food intake. As such, the eCB system is a highly conserved system among species, and the activation of the eCB system favors energy intake and conservation and inhibits energy expenditure [2]. In this regard, the eCB system regulates appetite, both centrally and peripherally, notably by controlling leptin and ghrelin signaling [3,4]. Thus, *Cnr*_1_-deficient mice are resistant to diet-induced obesity (DIO) while mice treated with CB_1_ receptor antagonists are leaner, due to a significant reduction in food intake [5,6]. In humans, the CB_1_ receptor inverse agonist Rimonabant was used to treat obesity to reduce appetite [7]. However, the drug was withdrawn few years later due to its side effects, notably on depression [1]. Herein, we review the roles and the complexities of the eCB system in the context of energy metabolism with an emphasis on adipose tissue metabolism.

## 2. Obesity

All living bodies, including *Homo sapiens*, have vital challenges: food availability and storage as well as reproduction. Since the 18th century, with the onset of the industrial revolution, drastic changes in lifestyle and food habits have occurred [8]. Thus, industrialization has gradually led to an overall obesogenic environment in which food consumption was higher than energy expenditure [9,10]. The word obesity is derived from the Latin words *obesitas*, meaning fatness, and *obesus*, which could be translated as ‘to eat all over debour’. Obesity mostly affects developed countries but also low- and middle-income countries. Beyond an excessive storage of energy, obesity is a state where the abnormal accumulation of fat leads to the deterioration of health. According to the World Health Organization, there are almost three million deaths associated with obesity every year [11]. This is because obesity is significantly associated with several chronic diseases such as type 2 diabetes [12], cardiometabolic disorders [13] and some types of cancer [14]. The body mass index (BMI) and waist circumference are the usual variables used to evaluate the health risk associated with obesity [15,16]. Waist circumference, in particular, can evaluate the presence of visceral obesity, thus helping to better predict the health alterations that come with obesity [17,18].

## 3. The eCB System

The eCB system is one of the most conserved system across vertebrate species [19,20]. By analyzing twelve phylogenetically diverse organisms, McPartland defined that eCB genes follow a heterogeneous evolutionary pathway, with functional orthologs [2]. Indeed, using phylogenetic tree analysis combined with phylogenomic comparison between vertebrates, invertebrates, bacteria, and archaea; McPartland found a conservative list of orthologs [21]. Furthermore, it has been proposed that the evolution of the eCB system results mostly from the coevolution between receptors and ligands [2,22].

The exploration of the eCB system in humans began with the discovery of the cannabinoid Δ9-tetrahydrocannabinol (THC) isolated from *Cannabis sativa* by Gaoni and Mechooulam in 1964 [23]. The psychoactive effect of THC suggested the presence of a receptor expressed in the central nervous system (CNS), which could bind the latter as well as endogenous ligands. The first documented cannabinoid receptor, the CB_1_, was cloned from a rat cerebral cortex cDNA library in 1990 [24]. Two years later, the second cannabinoid receptor, the CB_2_, was cloned from the promyeloid cell line HL-60 and found to be expressed by leukocytes, notably those arising from the myeloid precursor [25]. The CB_1_ and CB_2_ receptors are both G-protein-coupled receptors (GPCR) and share 44% of homology, and 68% of identity for the transmembrane domains [26]. According to the contrasting expression of these two receptors, it was initially assumed that CB_1_ is primarily expressed in the brain, while the CB_2_ receptor is mostly peripheral and expressed by immune cells. However, cannabinoid receptor expression is not a black and white story but rather a shade of greys. Indeed, several studies reported that the CB_1_ receptor is also expressed in peripheral tissues such as in testis [27,28], adipose tissues [29] or colonic tissues [30]. On the other side, the CB_2_ receptor was first documented as being absent from the CNS; however, CB_2_ mRNA and protein have been found in microglia, the resident immune cell of the brain [31,32,33,34]. Though CB_2_ is not abundant in microglia from heathy brain [35], its expression in these cells is upregulated (100 fold) during neuroinflammation and neurodegeneration diseases, such as experimental autoimmune encephalomyelitis, hypoxia/ischemia from middle cerebral artery occlusion and traumatic brain injury mice model [36,37,38,39].

The eCBs, AEA and 2-AG, are lipid mediators biosynthesized from cellular phospholipid membrane precursors. The biosynthesis of AEA can occur via different pathways although the cleavage of *N*-arachidonoyl-phosphatidylethanolamine by *N*-acyl phosphatidylethanolamine phospholipase D (NAPE-PLD) seems more widespread [40]. AEA signaling is then terminated by its metabolism by eicosanoid biosynthetic enzymes or by its hydrolysis by the fatty acid amide hydrolases (FAAH) [40]. In parallel, *N*-acyl-ethanolamine-hydrolyzing acid amidase, also known as NAAA, is considered as the second *N*-acyl-ethanolamine-hydrolyzing enzyme [41]. FAAH and NAAA have different activities, the former being a serine hydrolase localized in the endoplasmic reticulum and active in alkaline or neutral environment [42], while the latter is localized in lysosomes and active at acidic pH [43]. These enzymes can hydrolyze, with varying degrees of selectivity, *N*-acyl-ethanolamines to fatty acids (FAs) and ethanolamine [44]. As with AEA, 2-AG biosynthesis can occur via numerous biosynthetic pathways. In many cell types, 2-AG arises from the sequential actions of phospholipase C and 1,2-diacylglycerol (DAG) lipases [40], although an alternative and more efficient biosynthetic route exists in human leukocytes and involves the acylation of free FAs into cellular membranes, followed by a subsequent release of 2-AG with a possible LPA intermediate [45]. 2-AG is hydrolyzed by at least seven enzymes [46,47] although the most recognized one for this function is the monoacylglycerol (MAG) lipase [48]. AEA and 2-AG are not only endogenous ligands for CB_1_ and CB_2_ receptors [1,49,50], but also for transient receptor potential vanilloid type-1 (TRPV_1_) channels [49].

## 4. White Adipose Tissue (WAT)

The excess of consumed energy is mainly stored within the WAT, which is located in different parts of the body, but the most important fat depositions are the subcutaneous and visceral WAT (Table 1). In humans, the WAT is formed during gestation but in rodents, it develops only after birth. In rodents, the peri-gonadal fat depot is first developed, followed by the subcutaneous and omental depots [50]. Like muscle and bone cells, adipocytes are derived from mesenchymal stem cells [51]. Later, it has been reported that neural crest stem cells in culture are also able to differentiate into mature adipocytes upon simulation with growth factors and hormones [52]. In vertebrates, the stem cells from the neural crest can undergo an epithelial-mesenchymal transition and migrate to different regions where they undergo differentiation and hence, contribute to the formation of ectopic fat [52,53]. The maturation process of adipocytes was extensively studied in 3T3-L1 and 3T3-F422A mouse cell lines [54,55]. Farmer reviewed the network of transcriptional events which regulate adipocyte maturation and highlighted that PPARγ and C/EBPα are the two mains transcription factors for adipogenesis [53,56,57]. The exact number of white adipocytes is still under debate. There is a theory speculating that the number of adipocytes is defined during childhood and puberty [58]. Nevertheless, it was later demonstrated that adipocytes turnover is around 10% per year [59,60]. However, it is still conjectural whether adipocyte number increases during lifespan after preadipocyte division and differentiation, calling for further investigation. Adipocyte hyperplasia is associated with the early stage of adipocyte development during a premature stage of obesity, while hypertrophy occurs during tissue expansion, when adipocytes accumulate energy in the form of lipid vacuoles containing triglycerides [61,62].

## 5. Brown Adipose Tissue (BAT)

The BAT evolved in mammals to dissipate energy as heat to regulate body temperature by non-shivering thermogenesis (Table 1). In humans, the BAT is mainly present in the dorsal anterior and supraclavicular regions, as well as in a perirenal depot, deep neck region and around the spinal cord. In adult mice, the most important depot is in the dorsal anterior region [68,69]. The BAT is formed during embryogenesis to help the newborns to face the new cold environment and to become acclimatized since this tissue is activated by cold and helps to maintain the body temperature [70]. At the cellular level, brown adipocytes share the same embryonic precursors at myoblasts by expressing myogenic factor 5 (*Myf5*), which is a skeletal muscle marker of the mesoderm [71,72,73]. Cold induces the activation of sympathetic neurons to produce catecholamines, which can activate β3-adrenergic receptors on brown adipocytes. This stimulation drives the transcriptional responses in brown adipocytes and induces the expression and activation of uncoupling protein-1 (UCP1) [74]. UCP1 is present in the inner mitochondrial membrane and is activated by long chain FA produced within brown adipocytes post-lipolysis of cytoplasmic lipid droplets and upon systemic adrenergic receptors stimulation [64]. However, mice lacking lipid droplets and triglycerides in brown adipocytes maintain the normal body temperature [75]. It is suggested that brown adipocytes use FA derived from WAT to fuel the heat production [64,76]. As soon as it is activated, UCP1 can translocate protons (H+) from the intermembrane space to the mitochondrial matrix, disrupting the proton motive force needed by adenosine triphosphate (ATP) synthase to increase respiratory activity. Consequently, this leak of protons is converted to heat, reflecting energy expenditure, which is distributed all over the body through the blood circulation [77].

## 6. Involvement of the eCB System in Obesity

Cannabis has been used for millennia, starting from China and then moving west. In the early days, cannabis was called *ta ma* which means great hemp. It was principally a source of fiber for textiles, before it was used as a drug for recreational purposes and religious rites [78]. Before the Common Era, cannabis was utilized to treat rheumatism, gout, malaria, all diseases related to absent mindedness and, later, it was utilized during anesthesia [79]. Cannabis was also present in Indian medicine for sedative, relaxant, anxiolytic, anticonvulsant, appetite regulator, analgesic and antibacterial purposes [80,81]. The introduction of cannabis in Europe appeared in 19th century for medical and recreational use [82]. Interestingly, it is then reported that cannabis users have higher caloric intake compare to cannabis non-users [83,84]. However, Ngueta et al. observed that despite this high caloric consumption by cannabis users, they have a lower BMI [85]. In this regard, Clark et al. performed a meta-analysis comparing the BMI of cannabis users and non-users. This analysis confirmed that the BMI of obese cannabis users was lower compared to non-users by ~2 kg/m^2^ [86]. All these studies led to the concept that acute stimulation of CB_1_ by cannabis extracts initially increases food intake [87] while the overstimulation of the receptor by chronic consumption induces tolerance, which could be the consequence of CB_1_ internalization. This supports the concept that preventing CB_1_ signaling influences the energy metabolism in obesity and the idea of a dysregulated eCB system in obesity [88].

## 7. The eCB System Regulates the Inflammation Associated with the Adipose Tissue during Obesity

WAT and BAT contain immune cells that maintain and monitor the homeostasis of the tissue [89]. In lean adipose tissue, these immune cells have a type 2 immune response state. IL-33 secreted by the epithelial cells [90] induces the secretion of IL-5 and IL-13 by the resident innate lymphoid cells (ILC2s) [91]. IL-5 and IL-13 induce the activation of eosinophils which in turn secrete IL-4 which is a master regulator to maintain the resident macrophages in an alternatively activated state or M2-polarized phenotype [92]. M2 macrophages express several genes that encode anti-inflammatory cytokines such as IL-10 that help to maintain the insulin sensitivity of adipocytes [93]. In adipose tissue from obese patients, adipocyte hypertrophy results from excessive accumulation of FA within lipid vacuole. This morphological change leads to adipocyte hypoxia and oxidative stress [94] and results in the activation of transcription factors such as hypoxia-inducible factor 1 α (HIF-1α) [95]. As a consequence, stressed adipocytes start to secrete pro-inflammatory cytokines such as TNF-α, IL-6 and IL-1β [96]. Furthermore, adipocytes secrete chemokines including monocytes attractant protein-1 (MCP-1). The latter is strongly involved in the recruitment, but also the proliferation, of classically activated, or commonly named M1-polarized, macrophages [97,98,99]. Macrophages will in turn secrete pro-inflammatory cytokines that will maintain a pro-inflammatory environment in the adipose tissue, which will promote the polarization of M2 macrophages towards an inflammatory M1 phenotype [100]. This event is an important starting point in the establishment of metabolic diseases associated with obesity, including insulin resistance [101].

Both CB_1_ and CB_2_ receptors are involved in chronic inflammation associated with obesity [102]. In addition to progressive weight loss [103], chronic rimonabant administration is accompanied by a reduction in pro-inflammatory cytokines [104] and chemokines expression [105] in adipose tissue. Adipose tissue macrophages are an important source of inflammatory mediators [106]. For example, Miranville et al. showed that THP1-derived macrophages stimulated with LPS secrete pro-inflammatory cytokines, which in turn inhibited insulin receptor signaling in human adipocytes. However, when treated with CB_1_ receptor antagonist, these macrophages decrease the secretion of TNF-α and increase the level of IL-10 [107]. Furthermore, Han et al. observed that DIO and diabetic *db*/*db* mice treated with AJ5018, a CB_1_ receptor antagonist, for 4 weeks displayed lesser formation of crown-like structures around adipocytes and lower expression of immune cells markers in epididymal WAT [108]. A specific deletion of *Cnr*_1_ in adipocyte is sufficient to protect mice from DIO. These mice express more alternative macrophages markers in fat tissues. A flow cytometry analysis confirms an increase abundance of M2 macrophages in adipocytes-specific *Cnr*_1_-deficient mice [109]. To explore further this effect of CB_1_ antagonist on adipose tissue inflammatory status, Mehrpouya-Bahrami et al. investigated the role of micro-RNA in macrophage polarization and recruitment. They found that treatment with rimonabant altered the expression of several micro-RNA, particularly those that are members of the miR-466 family, which alter the expression of genes involved in M2 polarization [110]. CB_1_ receptor blockade with AM251 in mice fed with high fat diet upregulates miR-30e-5p particularly in macrophages and downregulates the DLL4-Notch signaling pathway, which is implicated in macrophage polarization [111].

Conversely, *Cnr*_2_-deficient mice fed with high fat diet were obese with high number of pro-inflammatory macrophages infiltrating the adipose tissue. In the same study, the authors showed that wild type mice treated with HU308, a CB_2_ agonist, have an inverse phenotype [112]. JWH-133, a CB_2_ receptor agonist, inhibits the activation of LPS-induced macrophages, and enhances the phenotype shift of M1 to M2 macrophages [113,114]. It can therefore be proposed that both receptors play key and opposite roles in the polarization of macrophages and in the regulation of chronic inflammation associated with obesity.

## 8. Link between the eCB System and Microbiota in the Context of Obesity

First of all, the gut microbiota influences the inflammation associated with obesity as high fat diet induces a shift toward a pro-inflammatory environment in gut [115]. Several evidences are emerging that the gut microbiota plays an important role in the genesis of obesity and related diseases induced by the low-grade inflammation associated with obesity [116]. The dysbiosis of the gut microbiota was linked to the regulation of the gut permeability and inflammation [117]. In addition, cannabinoid receptors are found in the colon [30] and their expression are increased in several inflammatory conditions such as celiac disease [118] and colonic inflammation [119]. For example, increasing the level of AEA and NAEs using the FAAH inhibitor PF3845 reduces inflammation induced by a 2,4,6-trinitrobenzene (TNBS) treatment [120]. Similarly, the increase of 2-AG prevents the systemic inflammation associated with colitis induced by TNBS [121]. A specific deletion of *Napepld* in adipocytes leads to increased levels of NAEs while the lack of this enzyme does not influence the AEA concentration. *Napepld*-deficient mice are obese and insulin resistant when fed with high fat diet. Furthermore, *Napepld* deletion altered the gut microbiota composition [122]. *Mgll*-deficient mice display high level of 2-AG, that protected mice from enteric infection with *Enterobacteriaceae* pathogens as 2-AG can inhibit directly the bacterial virulence [123]. In the context of obesity, targeting the CB_1_ receptor with rimonabant attenuated the gut permeability, modulated the expression of junction protein and inflammatory load, and resulted in an increased number of *Akkermansia muciniphila* [124].

Furthermore, gut microbiota influences the composition of hepatic and plasmatic FA [125]. Recently, it was found that the absence of microbiota alters the FAs content in the colon and the small intestine [126,127]. When they compared the expression of genes associated with eCB system including receptors and metabolic enzymes in small intestine, they found an alteration of gene expression in germ-free mice which were reversed by fecal microbiota transplant [128]. The deletion of *Mgll* in mice leads to an obesity-resistant phenotype that could be partially explained by changes in the microbiota [129]. Furthermore, the administration of *Akkermansia muciniphila* in DIO mice is associated with a higher levels of eCBs in the small intestine and helps improving the metabolic parameters associated with obesity [130].

The evidence of a link between the microbiota and the endocannabinoidome in human remains ill defined. However, the microbiota is sensitive to variations in body fat composition [131,132]. Castonguay et al. reported in human that dietary intakes of specific FAs were associated with 2-AG levels. Furthermore, the changes in the levels of 2-AG were associated with changes in *Peptostrptococcaceae, Veillonellceae* and *Akkermansiaceae* families [133]. Finally, a short- or long-term Mediterranean diet was associated with a lower circulating level of AEA and a higher fecal *Akkermansia mucuniphilia*, and this helped to improve insulin sensitivity and systemic inflammation [133,134].

## 9. The eCBs Are Modulated in Obesity

### 9.1. Animal Models

The biosynthesis of eCBs is enhanced by external stress such as metabolic challenges. As food quality influences eCB availability, mice fed with western diet enriched with AA and/or n-6 PUFA precursors demonstrated increased levels of AEA and 2-AG [93,135,136]. In an experiment conducted by Tara et al., mice subjected to a high fat diet for 9 weeks were reported having higher circulating levels of eCBs [137]. Then, overactivation of the eCB system associated with obesity stimulates increased caloric intake and reduced energy expenditure [138].

The local tissue concentration of eCBs is still under scrutiny as it is not yet clear if it can be reflected by the circulating level of eCBs. It is proposed that the eCB concentration in the adipose tissue is modified by the metabolic condition associated with an acute or a long-term high fat diet in mice [139,140]. However, Starowicz et al. raised the point that the eCB system modulation in this tissue could be associated with age rather than diet as the concentration of AEA and 2-AG are not different between mice fed with high fat diet when compared to the normal diet [141].

The variation of eCB concentrations in mice could be partly explained by eCB metabolic enzyme activities. Genetic deletion of *Dagla* in mice reduces the levels of 2-AG while the circulating level are not affected [142]. *Dagla*-deficient mice display the same phenotype as *Cnr*_1_-deficient mice regarding the eating behavior and the lean phenotype [143]. In addition, inactivation or genetic deletion of *Mgll*, which is a key enzyme in the hydrolysis of the 2-AG, results in an accumulation of 2-AG [144,145,146]. Furthermore, several groups have demonstrated that the absence of *Mgll* protects mice from DIO as well as from insulin resistance [147,148,149]. Most studies suggest that the phenotype associated with the absence of *Mgll* depends on its role in lipolysis. The lack of MAGL alters the availability of 2-AG since this enzyme hydrolyzes over 85% the latter [150]. However, the absence of cannabinoid receptor-related effects such as appetite stimulation, suggests a desensitization of cannabinoid receptors centrally [151,152,153] and peripherally [147] when 2-AG levels are elevated. In contrast, studies conducted by Chon et al. have shown that when *Mgll* is overexpressed specifically in small intestine, it results in reduced levels of 2-AG. In addition, mice were not resistant to DIO and showed increased appetite [154]. Furthermore, *Faah*-knockout mice display high levels of *N*-acylethanolamines [155]. The lack of *Faah* promotes the development of obesity as *Faah*-deficient mice fed with regular diet have higher body weight [156]. *Faah*-deficiency is associated with increased lipid storage and decreased insulin sensitivity [156,157].

### 9.2. Humans

Over the years, lipid consumption as well as composition has changed drastically [158]. The modern diet is composed of higher levels of omega-6 FA, with steep decrease in levels of omega-3 FA [144,159,160,161]. Linoleic acid and α-linolenic acid are the essential precursors of omega-6 and omega-3 families, respectively. Therefore, changing the percentage of dietary FA, such as linoleic acid, participates to modulate the bioavailability of eCB levels [161,162,163,164].

The changes in circulating levels of AEA and 2-AG associated with weight variation are one of the indicators of the perturbation of the eCB system [135]. Several studies report the absolute quantification of circulating eCB levels as well as the tissue local concentrations of eCBs in the context of obesity (Table 2 and Table 3). Most of the published data indicate that the circulating levels of 2-AG increase with BMI, and intra-abdominal obesity, suggesting that 2-AG is implicated in visceral fat accumulation [136,137,139,145,146,165,166,167]. However, Annuzzi et al. reported that the concentrations of 2-AG in subcutaneous adipose tissue is reduced by 2.3 folds in obese diabetic subjects [168]. Some other studies found that the levels of AEA are unchanged [82,83,92], while yet other reports show that AEA increases in obesity [166,169]. We hypothesize that this heterogeneity could be related to the lack of standardization in sample process and cohort choice. In addition, the heterogeneity could be explained by the analysis and research question leading to investigate the human cohorts in various ways, and considering different parameters such as gender and age, which can affect metabolic health. Importantly, the circulating levels of eCBs are reduced in viscerally obese men by weight loss induced by caloric restriction and exercise. The data from a post-bariatric surgery study suggest that the fluctuations being tightly associated with the metabolic status [170]. The variation of eCB concentrations could be partly explained by eCB metabolic enzyme activities. MAGL activity from human subcutaneous adipocytes does not show a link with BMI [171]. Others showed that the activities of DAGL and the MAGL in visceral and subcutaneous adipose tissues from obese patients are elevated [172]. FAAH activity in human subcutaneous adipocytes correlates with BMI and waist circumference [171,173]. More importantly, the synthetic and degrading enzyme activities are highly susceptible to genetic variations. A single nucleotide polymorphism (SNP) associated with *FAAH* (385C/A) results in a missense mutation and converts a conserved proline residue into threonine. This variant of *FAAH* does not alter the catalytic activity of the enzyme but increases its sensibility to proteolytic degradation [174]. Later, the gene coding for *FAAH* was compared between 115 obese subjects and 100 healthy subjects. The authors highlighted that the 385C/A polymorphism was associated with obesity. Indeed, obese subjects carrying this SNP have a significantly increased level of AEA and related NAEs [175,176]. In a study conducted by Sipe et al., 2667 subjects were genotyped for this polymorphism, and the homozygous *FAAH* A/A genotype was found to be a risk factor to develop obesity [177].

## 10. Targeting the eCB System to Treat Obesity

As we briefly introduced before, the eCB system influences energy balance by regulating food intake and energy expenditure. The former effect is due to modulation of both homeostatic and hedonic mechanisms and has been implicated also in eating disorders [191]. Drugs have been developed that target the endocannabinoid system, and particularly CB_1_ receptors, to modulate food intake and energy expenditure (Table 4).

### 10.1. Animal Models

Even though rimonabant was not used anymore in the clinic, several rodent studies with this drug have helped to understand the importance of the eCB system in obesity. Mice with DIO treated with SR141716 for 12 weeks present with an important reduction of obesity illustrated by a decrease of more than 50% of adipose tissue, which could be in part the result of the feeding reduction [5]. When mice are fed by daily oral gavage with high fat diet only for 30 days and then treated with rimonabant, they exhibit body weight reduction [103]. As this drug was developed for treatment purposes, 6 months-old obese mice were treated with rimonabant for 4 weeks. The first week of treatment caused an important food intake reduction accompanied by a drastic weight loss, which was maintained throughout the treatment [105,211]. Moreover, rimonabant or AM6545, a CB_1_ neutral antagonist, both improved the low-grade inflammation associated with obesity [39,131,212,213]. However, the effects of CB_1_ blockade to improve the metabolic syndrome are still under discussion. Migrenne et al. showed that rimonabant failed to improve insulin sensitivity in *adiponectin*-deficient mice, suggesting that CB_1_-mediated inhibition of adiponectin release might be an underlying mechanism for eCBs to cause insulin resistance [214]. In parallel, obese mice treated with rimonabant improved the lipid profile, which could be just the result of the negative energy balance induced by the treatment [215]. On the other hand, CB_2_ receptors are involved in the chronic inflammation associated with obesity as its expression increased with epididymal fat inflammation [201]. Deveaux et al. showed that insulin resistance associated with high fat diet was enhanced by treatment with the CB_2_ receptor agonist JWH-133 [201]. Insulin resistance and fat inflammation associated with obesity were improved by treatment with a CB_2_ receptor inverse agonist SR14528, but also in *Cnr*_2_-deficient mice [211,216,217]. These results suggest that CB_2_ receptor contributes to the obesity-associated metabolic disorder by regulating the chronic inflammation associated with obesity [112], which is counterintuitive considering that in other inflammatory conditions CB_2_ agonists have proven to be beneficial [218].

### 10.2. Humans

The first synthetic anti-obesity drug targeting the eCB system, i.e., rimonabant, also known as SR141716, was developed by Sanofi-Synthelabo Inc. It is a CB_1_ receptor-selective antagonist/inverse agonist [7,219]. As an anorectic drug in animals, rimonabant was used to reduce food intake and to treat obesity and related diseases, but also cocaine, heroin, nicotine and alcohol dependence. Rimonabant reached the clinical trial phase III for smoking cessation in 2003, while the phase III for obesity treatment had started on 2001 [219]. The effects of the treatment are consistent with the role of CB_1_ in facilitating energy accumulation and reward from substances of abuse. Several clinical studies have shown that rimonabant helps to reduce weight, and improves the metabolic parameters associated with obesity such as high waist circumference, low HDL-cholesterol, high triglycerides and, to a smaller extent, high blood pressure [192,193,220]. However, two years after its initial launch on the market, rimonabant was withdrawn due to side effects on mood, namely depression and anxiety [221]. Indeed, on the 5th November 2008, the company suspended its production and marketing for treatment [219]. Later, other strategies targeting the CB_2_ receptor were conceived, especially, to treat chronic inflammatory diseases such as Crohn’s disease or atopic dermatitis [121,222]. Adipocyte cultures obtained from human subcutaneous adipose tissue treated with AM630, which is a reverse agonist of CB_2_, decrease the secretion of adipokines. These results suggest that CB_2_ receptor is a potential therapeutic target for the treatment of obesity-related diseases [223]. As discussed before, it is also possible to modulate eCB availability and effects with certain short or long-term dietary supplementations. Two hours after an hedonic food consumption, there is a significant decrease of AEA and a significant increase of plasmatic levels of 2-AG [179]. A short term as well as a long-term diet modification with a Mediterranean diet is highly effective to increase the omega-3 PUFA–derived eCB-like mediators (NAEs and 2-MAGs), often at the expense of eCBs [133,224].

## 11. The eCB System and Peripheral Signals Controlling Food Intake

Leptin and ghrelin are two important peripherally-derived hormones that mediate satiety and hunger, respectively, and control, and are controlled by, eCBs [225].

### 11.1. Leptin

Identified by Zhang et al. in 1994, leptin is an appetite-suppressant cytokine produced by the adipose tissue [226]. Leptin receptor (LEP-R) is mainly expressed in brain areas involved in feeding regulation and energy expenditure, therefore, leptin enhances neuronal signaling in the hypothalamus as an anorexigenic mediator and promotes energy expenditure [212]. As overweight is the consequence of a positive energy balance and is accompanied by higher amounts of adipose tissue, it is not surprising to associate obesity with high circulating levels of leptin [213]. However, during obesity, leptin is not effective at reducing appetite and body weight. This is explained by leptin resistance, which is due to high leptin-induced inactivation of the LEP-R in the hypothalamus. Therefore, the therapeutic benefit of leptin treatment in obese subjects is still under debate [227]. There are several hypotheses related to LEP-R to explain leptin resistance [195]. The LEP-R signaling pathway is similar to cytokine signaling. When leptin binds to LEP-R, the conformational change induces the activation of janus kinase-2 (JAK2), which can phosphorylate tyrosine residues at the carboxyterminal tail of the homodimerized LEP-R. This induces the recruitment of signal transduces and activator of transcription 3 (STAT3). The phosphorylation of STAT3 is followed by its translocation into the nucleus. In turn, STAT3 initiates the transcription of suppressor of cytokine signaling 3 (SOCS3). SOCS3 is able to inhibit the LEP-R-JAK2 signaling pathway to provide a negative feedback to the LEP-R signaling [216]. A central suppression of SOCS3 in mice fed with high fat diet restores exogenous leptin sensitivity to reduce food intake and body weight [217]. Impaired leptin is associated with an over activation of the central eCB system [228,229]. As the eCB system can have an orexigenic effect, it is not surprising to see that the hypothalamic concentrations of eCBs are inversely correlated with the circulating levels of leptin [3]. CB_1_ receptor inactivation improves the response to intracerebroventricular leptin injection, and restores the leptin sensitivity to help to reduce obesity in a DIO mouse model [6]. In addition, when treated with a peripherally restricted CB_1_ receptor antagonist, JD5037, mice with DIO exhibit reduced leptin levels and subsequently restored leptin sensitivity, which leads to hypophagia [195]. Thus, both CB_1_ blockade and leptin show similar effects on appetite regulation [230]. As *Cnr*_1__-_knockout mice exhibit a reduced appetite, therefore, we can infer that the eCB system act as an appetite inductor through CB_1_ receptors. Di Marzo et al. revealed that rats treated with leptin had lower hypothalamic levels of AEA and 2-AG. In parallel, *ob*/*ob* mice deficient in leptin, and *db/db* mice deficient in leptin receptors, exhibit higher hypothalamic levels of eCBs compared to control C57BL/6J mice. Therefore, it is widely accepted that leptin plays a crucial role in controlling meal consumption via inhibition of hypothalamic eCB levels [3], suggesting a mutual regulation between leptin and eCB system.

### 11.2. Ghrelin

Ghrelin is secreted during negative energy state, when food availability is low, and decreased after food consumption [231]. It is not surprising to see that the serum levels of ghrelin are downregulated in obesity [232]. Ghrelin is secreted by enteroendocrine cells of the gastrointestinal tract, mostly by the stomach and the hormone is the first identified and the most studied orexigenic hormone from peripheral tissues [233,234,235]. Later, asprosin is identified to be secreted by the adipose tissue and induces an orexigenic effect in the central nervous system [236,237]. Focusing on the importance of ghrelin, the growth hormone secretagogue receptor has several isoforms but GHSR1a and GHSR1b are widely expressed [238,239]. Many of the endocrine functions of ghrelin is mediated by GHSR1a, identified as GHSR1, which is highly expressed in hypothalamic nuclei that regulates feeding and body weight [240]. In addition, the receptor is expressed by peripheral tissues such as the intestine, lung and pancreatic islets [241]. GHSR1 is a GPCR and forms a dimers and heterodimers with other GPCR to regulate feeding [242]. The activity of ghrelin depends on the activity of energy sensors such as sirtuin 1 (SIRT1) that can regulate energy balance [243]. Furthermore, GHSR1 stimulation results in the activation of (AMP)-activated protein kinase, which regulates pathways controlling glucose and FA metabolism as well as protein synthesis [244]. AMPK is also implicated in energy intake and body weight regulation as it acts as a metabolic sensor and promotes catabolic activity in peripheral tissues [245]. AMPK activation and inhibition mediate the action of orexigenic and anorexigenic signals, respectively [233,234,246]. Ghrelin can also activate phospholipase C followed by the generation of IP3 and diacylglycerol, thereby enhancing the increase of intracellular Ca^2+^. In turn, this can induce the stimulation of MAPK pathway to activate the cAMP response-element (CRE) to induce the transcription of the two orexigenic peptides neuropeptide Y (NPY) and agouti-related protein (AgRP) [234,247,248]. Mammalian target of rapamycin (mTOR), which is an intracellular nutrient sensor expressed in NPY/AgPR neurons is phosphorylated and activated by ghrelin [235]. The activation SIRT1, AMPK and mTOR pathways induces the activation of brain-specific homeobox domain (BSX), CREB and forkhead box1 (FOXO1) to increase the expression of NPY and AgRP to stimulate food intake [249].

The interaction between the eCB system and ghrelin has been investigated with several in vitro studies. The main role of the eCB system, and in particular of 2-AG, produced following PLCβ and DAGLα activation, and activating CB_1_ receptors, in ghrelin action is to mediate its orexigenic effects in the hypothalamus [250].

The link between the eCB system and ghrelin was also studied in animal models, although only 60% of double knockout for ghrelin receptor *Ghsr1* and *Cnr*_1_ survived after 20 weeks [4]. However, the absence of *Cnr*_1_ does not affect ghrelin availability [4]. This illustrates a mutual regulation between these two orexigenic signals. The central interaction between the eCB system and ghrelin to regulate food intake was deeply studied. Lim et al. showed that mice treated intraperitoneally with ghrelin or CB_1_ receptor agonist HU210 show an increase in AMPK activity in the hypothalamus. This AMPK activity induced by HU210 is abolished in mice deficient for the ghrelin receptor [251]. Therefore, the authors proposed that there is a mutual regulation between the eCB system and the ghrelin signaling pathways. This was confirmed by Kola et al. when they showed that the orexigenic signal of ghrelin is abolished in *Cnr*_1_-knockout mice and the AMPK activity induced by ghrelin is also inhibited by pharmacological blockade of CB_1_ [222,252,253].

Furthermore, the blockage of CB_1_ receptor by intraperitoneal injection of rimonabant can inhibit the secretion of ghrelin, however, an intracerebroventricular injection of rimonabant is not effective to reproduce the peripheral effect on ghrelin secretion [254]. A peripheral blockade of CB_1_ with rimonabant helps to reduce food intake but only in food-deprived animal. This could be the result of the decrease in the secretion of ghrelin following the activation of the mTOR/S6K1 signaling pathway in the stomach after the treatment with rimonabant [255]. A study conducted by Alen et al. demonstrated that mice treated with an intraperitoneal injection of rimonabant induces an anorexigenic effect in mice treated with an intracerebroventricular injection of ghrelin [256]. These studies suggested that there is a crosstalk between ghrelin and eCB system to modulate food intake, and this interaction can occur at a peripheral level.

## 12. The eCB System Regulates Adipose Tissue Function

### 12.1. WAT

As described above, the WAT is implicated in energy storage. In 2003, two independent groups pointed out the presence of CB_1_ in adipocytes suggesting that the eCB system is able to control adipocytes physiology [210,257]. Coherent with the importance of the eCB system in energy storage, several in vitro studies have demonstrated that CB_1_ activation in white adipocytes promotes adipogenesis by enhancing the formation of triglyceride-rich lipid droplets [258]. In addition, *Cnr*_2_ is also expressed by preadipocytes and mature adipocytes from visceral and subcutaneous adipose tissue. As both receptors are expressed by adipocytes, these cells are likely to be sensitive to THC [139].

#### 12.1.1. Animal Models

Several studies investigated the role of the eCB system in adipocyte hypertrophy and hyperplasia, the two most important mechanisms to which adipocytes undergo to accumulate energy excess. Matias et al. found that 3T3-F442A adipocytes treated with HU210, a CB_1_ receptor agonist, enhance the expression of PPARγ to favor lipid droplet accumulation [259]. Moreover, AEA can act through the receptor PPARγ, amplifying the importance of this eCB in adipogenesis [260]. Rimonabant can target the WAT and this treatment results in a strong reduction in white adipocyte size. The number of adipocyte nuclei observed in the WAT of treated mice suggests that the weight loss associated with rimonabant administration is a result of adipose tissue reshaping rather than increased energy loss [5]. However, Gary-Bobo et al. described that rimonabant inhibits 3T3-442 preadipocyte proliferation in a concentration-dependent manner and by inhibiting MAP kinase activity. Furthermore, rimonabant treatment enhanced the expression of genes involved in adipocyte maturation at the highest concentration of the drug, while there was no visible increase in lipid droplet accumulation [208], possibly suggestive of a non-CB_1_-mediated effect at high doses.

In parallel, the eCB system plays an important role in adipocyte differentiation via both CB_1_ and PPARγ dependent mechanisms [261]. AEA can act through these receptors to induce the differentiation and lipid accumulation of 3T3-L1 cells [209,260]. A study from Wagner et al. indicates that the absence of *Cnr*_1_ accelerates the differentiation of subcutaneous adipocytes from *Cnr*_1_-knockout mice [262], again suggesting non CB_1_-mediated effects. However, several studies support that CB_1_ is implicated in promoting adipocyte differentiation and maturation. CB_1_ stimulation induces the activation of LPL and promotes lipogenesis by stimulating triglyceride biosynthesis by inhibiting AMPK. Δ^9^-THC stimulation reduces AMPK activity in visceral and subcutaneous adipose tissues, which causes enhancement of FA synthesis [199,207]. Therefore, it can be suggested that the eCB system can influence the adipose tissue to adapt to energy excess [263].

#### 12.1.2. Humans

Human white subcutaneous adipocytes are a significant source of AEA and 2-AG [185]. Spoto et al. affirmed that the human adipose tissue is able to respond to AEA and 2-AG because it expresses the two cannabinoid receptors [184]. In fact, differentiated adipocytes show higher *CNR_1_* and *FAAH* mRNA expression levels compared to undifferentiated cells [4]. On the other hand, *CNR_2_* is also expressed by preadipocytes, although its expression does not change during adipocyte differentiation [29]. Ruhl et al. used mesenchymal stromal cells from human subcutaneous adipose tissue, which they stimulated with the CB_1_/CB_2_ agonist WIN55,212-2, and the specific CB_2_ agonist JWH-133, along with rimonabant or AM630 (a CB_2_ inverse agonist). They demonstrated that CB_1_ stimulation decreases human adipocyte metabolic activity and the cell division, which is reversed by treatment with rimonabant [263].

It is known that the visceral adipose tissue presents with higher metabolic activity compared to the subcutaneous adipose tissue [4,247,248]. The eCB system has been scrutinized in human adipose tissue and compared between the visceral and subcutaneous adipose tissues. It has been reported that the mRNA expression of *CNR_1_* receptors is higher in visceral adipose tissue from obese subjects compared to their subcutaneous adipose tissue [92,192,264]. Blüher et al. also confirmed that the mRNA expression of *FAAH* is higher in visceral adipose [166]. However, the expression of *MAGL* mRNA is higher in the subcutaneous adipose tissue [172]. Even though the gene expression of these two critical eCB catabolic enzymes were different from one depot to the other, the activity of FAAH and MAGL was not different in isolated adipocytes from visceral or subcutaneous adipose tissue [265].

### 12.2. BAT

As described above, the BAT is implicated in energy dissipation by heat, and it is implicated in body temperature regulation in response to cold exposure [64]. The non-shivering thermogenesis promoted by BAT is enhanced by the activation of the sympathetic nervous system and activates β-adrenoreceptors after catecholamine release. The WAT is also sensitive to β-adrenergic stimulation after prolonged cold exposure [266]. Under β-adrenergic stimulation, white adipocytes become brown-like cells. This intermediate phenotype is known as beige or brite adipocyte. The browning process was described for the first time by Young et al. in 1984 [252]. During this process, white adipocytes start to express UCP1 and initiate to acquire brown adipocyte characteristics such as the increased number of mitochondria and small lipid droplets, until they form the beige adipocytes [72].

Several human studies focusing on BAT metabolism were conducted by Carpentier et al. by using PET/CT imaging. They confirmed that the human BAT is sensitive to β-adrenergic stimulation and proposed BAT stimulation with pharmacological agents as a target to improve the metabolic status [253,267,268,269]. However, even though human thermogenesis is a way to lose weight, it helps mostly to maintain the weight loss and improve the metabolic syndrome associated with obesity [267]. Primary brown adipocytes and BAT from mice treated with the β3-adrenergic receptor agonist CL316,243, expressed higher level of eCBs and synthetic enzymes suggesting that the eCB system can be regulated by a signal triggering the activation of energy expenditure by thermogenesis [189]. By investigating the importance of the eCB system in the browning process during obesity, studies in mice showed that genetic depletion of *Cnr*_1_ enhances energy expenditure by the BAT [109]. This specific deletion of *Cnr*_1_ in the adipose tissue is sufficient to maintain a lean phenotype and enhanced energy expenditure in mice also following a high fat diet [109]. The importance of CB_1_ as a peripheral receptor in thermogenesis was reinforced by the use of BPR0912, a CB_1_ receptor antagonist, which enhanced the WAT browning process [196]. Conversely, the activation of CB_1_ with its synthetic agonist, arachidonyl-2-chloroethanolamide, suppresses mitochondrial biogenesis, which favors the white adipocyte phenotype at the expense of the browning process [139,270]. Additionally, *Cnr*_1_ gene deletion in the forebrain and sympathetic neurons can also control the thermogenesis. A study performed in brain *Cnr*_1_-deficient mice showed an increased number of mitochondria and higher thermogenesis in BAT [194]. All these studies suggest that the eCB system has an inhibitory effect on thermogenesis, which strengthens its role in energy conservation.

## 13. The eCB System: Biomarker or Treatment

According to the evidence reviewed and summarized above, it is evident that the eCB system is implicated in energy metabolism centrally and peripherally (Table 5). CB_1_ receptor can also act through the sympathetic nervous system to induce the hypophagia associated with CB_1_ receptor blockade. In addition, peripheral blockade of sympathetic neurotransmission can abolish the central effect of CB_1_ blockade. Authors suggested that CB_1_ receptor can act centrally and peripherally through the activity of peripheral sympathetic activity [271].

Several metabolic mechanisms involve the peripheral CB_1_ as reflected by the effects of CB_1_ blockers suggesting the pleiotropic role of this receptor on energy metabolism. Before rimonabant was withdrawn, its effects on endocannabinoids, adipocytes, liver, skeletal muscle functions linked to systemic glucose intolerance and insulin resistance had already been well explored [272,273,274]. Therefore, a strategy targeting a selective antagonism of CB_1_ receptor had started. Until now, JD5037 which is a CB_1_ blocker, shows all the positive effects including full specificity for CB_1_ with low brain penetration [275] and has a beneficial effect on DIO mice [276]. Several drugs targeting either the cannabinoid receptors or the inhibition of the catabolic enzymes FAAH or MAGL were tested regarding their potential benefits in obesity and associated diseases [1]. For example, the inhibition of FAAH is mostly for treating pain and CB_2_ agonist is used to treat chronic inflammatory diseases [277,278,279]. Nonetheless, so far, the results remain ambiguous.

It is more and more evident that the overactivation of the eCB system is a critical feature associated with obesity and metabolic disorders [146]. Therefore it is interesting to explore the eCBs as a biomarker to predict the metabolic disorders associated with obesity [178]. A study performed in a large cohort comparing lean and obese subject demonstrated that changes in plasma levels of 2-AG could help to predict the occurrence of cardiometabolic diseases such as dyslipidemia and insulin resistance in lean aged men while the levels of 2-AG correlate with metabolic diseases in women in menopause, although it is not associated with BMI [280]. However, in order for a parameter to be a good candidate as biomarker, it is important that the biological sample wherein the parameter is measured is easy to access and analyze. Therefore, it is advisable to quantify eCBs in either blood or saliva, depending on the outcome profile. The levels of circulating eCBs in plasma of obese patients could be used to predict the risk factor associated to obesity [178]. In addition, some studies demonstrate that the concentration of eCBs in saliva change according to the body weight gain or loss. Both AEA and 2-AG are readily quantifiable in saliva, and their levels are significantly high in obese subjects and decreased after weight loss [281]. However, several parameters can affect the composition of saliva but also the composition of eCBs. We can mention among many others that lipolysis and proteolysis activities of saliva differ between obese and lean people [264]. In addition, the composition of saliva can be affected by the lifestyle as the concentration of eCBs are readily modified under conditions such as mastication or stress generated by a physical activity [282,283].

In conclusion, this review has highlighted the importance of the eCB system in the regulation of metabolism. Furthermore, the dysregulation of this system could become an alarm signal for excessive energy balance. In the meantime, the understanding of the mechanisms involving the eCB system in various processes such as the immune response will allow the sharpening of therapeutic targets not only in metabolic diseases but also in other chronic inflammatory diseases. Further investigations of the role of the eCB system on the regulation of energy metabolism could result in new mechanisms, components and roles of eCBs and eCB-like molecules, which would lead to the identification of new targets for the treatment of obesity and metabolic disorders.

## Figures and Tables

**Table 1:** Adipose tissue biology. Overview of the comparative characteristics of white (WAT) and brown (BAT) adipose tissue and their respective roles. Myf5, myogenic factor 5; UCP1, uncoupling protein 1.

	WAT	BAT
Function	Energy storage [63]	Heat production (nonshivering thermogenesis) [64]
Origin	From mesoderm and neural crest	From mesoderm *Myf5* positive progenitor
Morphology	Single lipid droplet [53]	Multiple small vacuoles, abundant mitochondria High vascularization [64]
Characteristic protein	Leptin and adiponectin [65]	UCP1 [53,64]
Activation	Intensive physical activity Hormonal stimulation after starvation [66]	Cold β-adrenergic stimulation by catecholamines [64]
Development	Increase with age and body weight [63]	Abundant in new-born and decrease with age [67]

**Table 2:** Circulating level of endocannabinoids associated with obesity. AEA and 2-AG are detected in the plasma and are modulated according to the metabolic condition. DIO, diet-induced obesity.

Model	Model	AEA	2-AG	References
Human	Obesity	↑	↑	[146,178]
	Obesity	↑	↓	[179]
	Hedonic eating	↓	↑	[179]
	Weight loss after bariatric surgery	↓	↓	[170]
	Caloric restriction	↓	↔	[180]
	Anorexia	↑	NA	[181]
	Prader-Willi syndrome	↑	↑	[182]
	Diabetes	↑	↑	[139]
Mice	DIO	↑	↑	[137]
	DIO	↔	↑	[146,166]
	Western diet	↑	↑	[183]

↑, increasing; ↓, decreasing; ↔, no change.

**Table 3:** Circulating level of endocannabinoids associated with obesity. AEA and 2-AG detected in various tissues are modulated according to the metabolic condition. DIO, diet-induced obesity.

Model	Model	Tissue	AEA	2-AG	References
Human	Obesity	Visceral WAT	↑	↑	[184]
	Obesity	Visceral WAT	↔	↑	[139]
	Obesity	Subcutaneous WAT	↑	↑	[185]
Mice	DIO	Small intestine	↑	↓	[186]
	DIO	Small intestine	↑	↑	[138]
	Food deprivation	Small intestine	↑	↑	[187]
	DIO	Visceral WAT	↑	↑	[141,188]
	DIO	Subcutaneous WAT	↑	↑	[83,89,100]
	DIO	Subcutaneous WAT	↑	↓	[140]
	CL316, 243	BAT	↑	↑	[189]
Rats	Zucker rats	duodenum	↓	↑	[186,190]
	Zucker rats	Liver	↑	↑	[186]

↑, increasing; ↓, decreasing; ↔, no change.

**Table 4:** Effect of treatments targeting more or less selectively the eCB system. Several treatments are being developed, which can influence metabolism or the inflammatory response, to treat obesity and associated metabolic diseases. DIO, diet-induced obesity; FA, fatty acid; PUFA, poly-unsaturated fatty acids.

	Model	Treatment	Function	Effects	References
Human	Obesity	Rimonabant (SR141716)	CB_1_ inverse agonist	↓ food intake ↓ waist circumference improve metabolic parameters	[192,193]
	Obesity	Cannabis	Cannabinoid receptor agonist	↑ food intake ↓ weight	[83,85,194]
Mice	DIO	Rimonabant (SR141716)	CB_1_ inverse agonist	↓ obesity ↓ adiposity ↓ food intake ↓ adipocytes size ↑ adipocytes number (hyperplasia) ↑ macrophage M2 polarization	[5,110,124]
	DIO	AJ5012	CB_1_ antagonist	↓ body weight ↓ inflammation	[140]
	DIO	JD5037	CB_1_ antagonist	↑ leptin sensitivity ↓ body weight improvement of glucose and lipid metabolism	[195]
	DIO	AJ5012	CB_1_ antagonist	↓ body weight ↑ energy expenditure ↑ insulin sensitivity ↓ adipose tissue inflammation	[140]
		BPR0912	CB_1_ antagonist	↓ body weight ↑ β-oxydation and thermogenesis	[196]
	Monosodium glutamate (MSG)-induced hypometabolic and hypothalamic obesity	AM6545	CB_1_ antagonist	↓ body weight no effect on food intake ↑ circulating adipokine ↓ inflammation	[197]
	DIO	Pregnenolone	Allosteric inhibitor	↓ body weight ↓ food intake	[198]
	DIO	∆^9^-THC	Cannabinoid receptor agonist	↓ AMPK activity ↑ adipogenesis	[199]
	DIO	2-AG	CB_1_ and CB_2_ agonist	↑ insulin sensitivity ↑ AKT phosphorylation and GLUT4 translocation	[200]
	*ob/ob*	JWH-133	CB_2_ agonist	↑ adipose tissue inflammation ↑ insulin resistance	[201]
Rats	DIO	AM630	CB_2_ antagonist	weight gain blockage	[202]
	DIO	Cannabidiol	CB_1_ and CB_2_ agonist	↓ weight loss	[202]
	DIO and chow-fed rats	PSNCBAM-1	Allosteric inhibitor	↓ body weight ↓ food intake	[203]
	Zucker rats	Diet enriched in n-3 PUFA	FA	↓ level of endocannabinoid in adipose tissue, liver ↓ ectopic fat ↓ inflammation	[204]
	Cold exposure	Rimonabant (SR141716)	CB_1_ inverse agonist	↑ 1.5 to 5 °C of body temperature ↓ Body weight	[205]
	Diet enriched in n3-PUFA	n3-PUFA-enriched food	FA	↓ LDL cholesterol ↓ waist/hip ratio ↓ visceral fat/skeletal muscle mass ↓ AEA	[163,206]
In Vitro	3T3-F442	HU210	CB_1_ antagonist	↑ PPARγ expression	[207]
	3T3-F442	Rimonabant (SR141716)	CB_1_ inverse agonist	↓ proliferation ↑ maturation ↓ MAPK pathway	[208]
	3T3-L1	AEA	CB_1_ and CB_2_ agonist	↑ lipid accumulation	[209]
	3T3-L1	Insulin		↓ AEA ↓ 2-AG	[175]
	Mice adipocytes	WIN-55, 212	CB_1_ agonist	↑ lipogenesis	[210]
	Mice adipocytes	AEA	Transcriptional activation of Pparγ	Adipocytes differentiation and lipid accumulation	[209]

↑, increasing; ↓, decreasing; ↔, no change.

**Table 5:** Summary of the major effects of eCB system on peripheral tissues. The eCB system promotes the conservation and intake of energy by targeting key cellular mechanisms in adipose tissues. It also acts on several metabolically active organs to control glucose and lipid metabolism.

Organ	Effects
Brain and CNS	↓ Energy expenditure and BAT thermogenesis [189,284] ↓ gastrointestinal motility via the vagus nerve [285] ↑ hedonism (food-seeking behaviour) [191] ↓ gastrointestinal motility via the vagus nerve [286]
Gastrointestinal tract	↑ ghrelin secretion [287] ↓ motility [286,288] ↑ nutrient absorption with fat preference and intake [289] ↑ insulin secretion by the pancreas [141] ↑ lipogenesis by the liver [290] ↓ insulin clearance via PI3-kinase and calcium dependent mechanism [291,292]
White adipose tissue	↓ leptin secretion [195] ↓ lipolysis [273] ↑ storage capacity: ↑adipogenesis and ↓ preadipocytes [263]
Brown adipose tissue	↓ thermogenesis [189]
Skeletal muscle	↓ glucose uptake [293] ↓ insulin signalling [293,294]

↑, increasing; ↓, decreasing; ↔, no change.

## Data Availability

Not applicable.
